# Beyond the Bruise: Unmasking Disseminated Intravascular Coagulation in Alcohol-Related Presentations

**DOI:** 10.7759/cureus.93352

**Published:** 2025-09-27

**Authors:** Sri Amarnath Mathiyalagan, Aemon Fatima, Syeda Safa Sadiya, Lunik Sarder

**Affiliations:** 1 Emergency Medicine, Barking, Havering and RedBridge University Hospitals National Health Service (NHS) Trust, London, GBR

**Keywords:** alcohol intoxication and trauma, alcohol-related liver disease (arld), bruising differential diagnosis, cirrhotic coagulopathy, diagnostic overshadowing, disseminated intravascular coagulation (dic), isth dic score, overt dic vs cirrhotic coagulopathy

## Abstract

Disseminated intravascular coagulation (DIC) is a rare but life-threatening complication encountered in the emergency department (ED). It results from widespread activation of coagulation pathways, leading to simultaneous thrombosis and consumption of clotting factors, often culminating in severe bleeding and multi-organ failure. We report the case of a 39-year-old female with alcohol-related cirrhosis and autoimmune hepatitis who presented to the ED with a minor head injury and active bleeding from a groin ulcer. Her initial evaluation was confounded by alcohol intoxication and suspicion of domestic violence due to multiple bruises. Only after a delayed laboratory assessment was a diagnosis of DIC confirmed in the context of decompensated chronic liver disease with hepatic encephalopathy. She was treated with blood products and discharged with follow-up in the hepatology department. This case illustrates the diagnostic challenges of distinguishing trauma-related bruising from systemic coagulopathy in alcohol-related presentations. It emphasizes the importance of maintaining a high index of suspicion for DIC in cirrhotic patients, the role of the International Society on Thrombosis and Haemostasis DIC score, and the risks of diagnostic overshadowing in the ED. Early recognition and multidisciplinary intervention can significantly improve patient outcomes.

## Introduction

Disseminated intravascular coagulation (DIC) is an uncommon but critical hematological emergency. It is characterized by systemic activation of coagulation, resulting in widespread microvascular thrombosis, consumption of platelets and clotting factors, and an increased risk of life-threatening bleeding [1]. The incidence of DIC is approximately 1% in hospitalized patients and as high as 30-50% in patients with septic shock [1,2]. Trauma, malignancy, and obstetric catastrophes are well-established precipitants [1,3,4]. Patients with advanced liver disease are uniquely challenging: cirrhosis produces a fragile but rebalanced hemostatic state that mimics or masks DIC, since both conditions are associated with thrombocytopenia, prolonged coagulation times, and abnormal fibrinogen levels [3,5]. Distinguishing between cirrhotic coagulopathy and overt DIC is essential, as the latter carries a much higher risk of rapid decompensation and poor outcomes. This case report describes a woman with alcohol-related cirrhosis who presented to the emergency department (ED) after a fall, with widespread bruising initially attributed to trauma and possible domestic violence. The eventual diagnosis of overt DIC illustrates the risks of diagnostic overshadowing in patients with alcohol dependence. It highlights the importance of structured tools such as the International Society on Thrombosis and Haemostasis (ISTH) DIC score in guiding early recognition.

## Case presentation

A 39-year-old female with a background of alcohol dependence, alcohol-related cirrhosis, and autoimmune hepatitis presented to the ED with minor head trauma and active bleeding from a left-sided groin ulcer. She arrived under the influence of alcohol, which limited the reliability of history-taking. Examination revealed multiple bruises across her forearms and trunk, raising concerns about possible domestic violence. On regaining sobriety, she firmly denied assault as the cause of her bruising. On examination, she was pale, icteric, and had widespread ecchymoses. A bleeding, ulcerated groin ulcer and asterixis were noted. She was clinically assessed as having decompensated chronic liver disease with hepatic encephalopathy stage 1, and CT head reported as no acute concerns. The differential diagnoses included trauma-related soft tissue injury, coagulopathy secondary to liver dysfunction, and systemic hematological disorders such as DIC. Initial laboratory investigations are summarized in Table 1.

**Table 1:** Key laboratory findings on admission

Test	Result	Reference range
Hemoglobin	67 g/L	115-155 g/L
Platelets	50 × 10⁹/L	150-400 × 10⁹/L
Prothrombin time	38.2 sec	9-13 sec
Activated partial thromboplastin time	51.2 sec	20-33 sec
Fibrinogen (Clauss)	0.30 g/L	1.5-4.0 g/L
D-dimer	19.69 mg/L FEU	<0.50 mg/L FEU
Total bilirubin	122 µmol/L	1-21 µmol/L

These results fulfilled the ISTH criteria for overt DIC [1]. She was managed with one unit of packed red blood cells, five units of cryoprecipitate, vitamin K, and supportive care. Her fibrinogen levels improved gradually to 1.08 g/L over the course of a four-day admission. Other than bruising, she had no further overt bleeding. After stabilization, she was discharged with a referral to hepatology for ongoing management. Serial laboratory monitoring demonstrated gradual improvement in coagulation parameters, as shown in Table 2.

**Table 2:** Trend of key laboratory results during admission

Test	Day 1	Day 3
Hemoglobin	68 g/L	78 g/L
Platelets	67 × 10⁹/L	76 × 10⁹/L
Prothrombin time	18.8 sec	16.2 sec
Activated partial thromboplastin time	38.5 sec	33.6 sec
Fibrinogen (Clauss)	0.80 g/L	1.03 g/L

## Discussion

Epidemiology and presentations

This case underscores the importance of distinguishing trauma-related bruising from systemic coagulopathy such as DIC, particularly in patients with alcohol-related liver disease. Alcohol misuse frequently results in ED presentations with falls, injuries, and bruising [1]. Baseline cirrhotic coagulopathy further complicates the clinical picture. However, cirrhosis alone does not equate to DIC; rather, it predisposes patients to DIC when combined with systemic stressors such as infection, trauma, or decompensation [3,5].

DIC occurs in ~1% of hospitalized patients, up to 50% in septic shock, and 20% of malignancy-related admissions [1,2,6-8]. In obstetrics, the incidence is 2-3 per 1000 deliveries [4]. In trauma, DIC doubles mortality rates [2]. Case reports describe diverse presentations, including thyroid storm [6], heatstroke [7], and occult malignancy [8], underscoring the protean nature of DIC. Purpura fulminans, a catastrophic dermatologic manifestation, exemplifies the end of this spectrum [9].

Cirrhosis and DIC

Stable cirrhosis is not synonymous with DIC, but cirrhotic patients are particularly vulnerable when stressed. Chronic liver disease produces a rebalanced but fragile hemostatic state [3,5]. As a result, cirrhotic patients may have abnormal coagulation tests (prolonged PT/INR, thrombocytopenia) yet remain in relative balance. When acute triggers such as infection, bleeding, or trauma occur, this balance tips rapidly into overt DIC [3].

Cirrhotic coagulopathy versus overt DIC

Cirrhotic patients often present with abnormal hemostatic parameters, making the diagnosis of DIC particularly challenging [3,5]. Several laboratory differences have been proposed to aid in the distinction. Fibrinogen levels are often normal or elevated in stable cirrhosis, whereas they are typically low in overt DIC. Factor VIII is typically elevated in cirrhosis as an acute-phase reactant; however, in DIC, it becomes reduced due to consumption. D-dimer levels may be only mildly elevated in cirrhosis, while they are markedly elevated in DIC. Finally, the dynamic behavior of coagulation tests provides another clue: in cirrhosis, derangements remain relatively stable, whereas in DIC, laboratory values worsen rapidly over hours [3,5,10].

The ISTH DIC score has been validated for critically ill patients and enjoys global acceptance [1,2]. However, its accuracy in diagnosing cirrhosis is debated [3,5]. In such cases, trends in fibrinogen and platelets, together with clinical context, are essential to avoid overdiagnosis [5].

## Conclusions

This case highlights the diagnostic challenge of distinguishing DIC from cirrhotic coagulopathy in the ED. In a patient with alcohol dependence, liver cirrhosis, and trauma, diagnostic overshadowing initially delayed recognition of life-threatening DIC. The patient’s presentation with severe hypofibrinogenemia, high D-dimer, and worsening coagulation profile fulfilled ISTH criteria for overt DIC, demonstrating how dynamic changes differentiate DIC from stable cirrhotic coagulopathy. Early recognition and intervention, combined with supportive blood products, led to stabilization and a safe discharge. Clinicians must maintain a high index of suspicion for DIC in cirrhotic patients presenting with unexplained bruising or bleeding, even when trauma or alcohol intoxication provides alternative explanations. This case highlights the importance of structured scoring systems, timely coagulation testing, and multidisciplinary management in enhancing outcomes.
